# Prediction of coffee aroma from single roasted coffee beans by hyperspectral imaging

**DOI:** 10.1016/j.foodchem.2021.131159

**Published:** 2022-03-01

**Authors:** Nicola Caporaso, Martin B. Whitworth, Ian D. Fisk

**Affiliations:** a Division of Food Sciences, University of Nottingham, Sutton Bonington Campus, LE12 5RD, UK; b Campden BRI, Chipping Campden, Gloucestershire GL55 6LD, UK; c The University of Adelaide, North Terrace, Adelaide, South Australia, Australia

**Keywords:** Hyperspectral chemical imaging, NIRS, Coffee aroma, Flavour development, Quality control, Coffee roasting, Non-destructive assessment

## Abstract

Coffee aroma is critical for consumer liking and enables price differentiation of coffee. This study applied hyperspectral imaging (1000–2500 nm) to predict volatile compounds in single roasted coffee beans, as measured by Solid Phase Micro Extraction-Gas Chromatography-Mass Spectrometry and Gas Chromatography-Olfactometry. Partial least square (PLS) regression models were built for individual volatile compounds and chemical classes. Selected key aroma compounds were predicted well enough to allow rapid screening (R^2^ greater than 0.7, Ratio to Performance Deviation (RPD) greater than 1.5), and improved predictions were achieved for classes of compounds - e.g. aldehydes and pyrazines (R^2^ ∼ 0.8, RPD ∼ 1.9). To demonstrate the approach, beans were successfully segregated by HSI into prototype batches with different levels of pyrazines (smoky) or aldehydes (sweet). This is industrially relevant as it will provide new rapid tools for quality evaluation, opportunities to understand and minimise heterogeneity during production and roasting and ultimately provide the tools to define and achieve new coffee flavour profiles.

## Highlights

• This paper applied hyperspectral imaging (HSI) to predict roasted coffee aroma profile.
• Individual roast coffee beans were analysed by HSI and aroma by GC–MS.
• PLS models successfully predicted volatile aroma compounds in single coffee beans.
• Beans were successfully segregated into two batches with different aroma profiles.

## 1 Introduction

Coffee is a highly traded agricultural commodity. Its price and quality are strongly dependent on the aroma obtained after roasting and brewing. Coffee aroma is therefore of great commercial interest and important for the enjoyment of the product by the consumer. The volatile composition of roasted coffee is complex, with more than 800 compounds identified. Whilst many attempts have been made to link the complex pattern of coffee volatile compounds to its sensory quality, the selection of “key aroma compounds” is most commonly used to identify compounds that contribute to the overall aroma (Sunarharum, Williams, & Smyth, 2014). However, coffee batches are not homogeneous and, in many cases, contain both within specification coffee beans and beans with unique flavour profiles.

Coffee aroma is generated during roasting, which causes a complicated pathway of reactions (Farah, Monteiro, Calado, Franca, & Trugo, 2006), that can be both positive and negative (Giacalone et al., 2019, Yang et al., 2016). These include the Maillard and Strecker reaction and degradation of proteins, sugars, trigonelline and chlorogenic acid (De Maria, Trugo, Neto, Moreira, & Alviano, 1996). Different types of coffee beans, including different varieties, geographical origins or post-harvest treatments, can lead to different aromas, even when they are roasted under identical conditions.

It is generally accepted that a medium roasted coffee contains the highest amount of volatile compounds (Yeretzian, Jordan, Badoud, & Lindinger, 2002), and that a light roasted coffee gives rise to stronger notes of sweet, cocoa and nutty aroma, while a darker roast causes more intense notes of burnt, sour, pungent and roasted aroma (Bhumiratana, Adhikari, & Chambers, 2011). Examples of key aroma volatiles identified in roasted and ground coffee beans include 2-ethyl-3,5-dimethylpyrazine, 3-mercapto-3-methylbutyl-formate, 2-furfurylthiol and β-damascenone.

In addition to analysing variation in volatile compounds at a batch level, it is also relevant to look at variation within batches, including for individual coffee beans, but there have been relatively few studies of this. Caporaso et al. (2018) analysed the flavour profile of single beans by SPME GC–MS and reported a wide variation between beans even within the same batch, which depended on the chemical class of the volatile compounds. Fischer et al. (2014) used time-of-flight mass spectrometry (TOF-MS) to evaluate volatile release over time during roasting. The authors were tentatively able to link the volatile compounds generated to the possible precursors. Pyridines were reported to be generated from trigonelline, phenols from chlorogenic acids, furfuryl alcohol from carbohydrates, and 1,2-benzenediol, guaiacol, vinylguaiacol and ethylguaiacol from chlorogenic acids. This was further built on by Liu et al. (2019) through the addition of targeted flavour precursors to modify coffee aroma. However, further research is still required to fully understand volatile compound formation in coffee, and to link its presence to flavour precursors in green coffee (Dorfner, Ferge, Kettrup, Zimmermann, & Yeretzian, 2003).

Despite the numerous analytical techniques available to measure the volatile composition of coffee beans, most are time-consuming and some use harmful chemicals. Near-Infrared (NIR) spectroscopy (NIRS) has been used for the evaluation of espresso coffee brew quality, by trying to correlate the spectral information to the sensory response of a panel of consumers (Esteban-Diez, González-Sáiz, & Pizarro, 2004). Good prediction of several sensory attributes by NIRS was reported, with the best performance of R^2^ = 0.9 being achieved for perceived acidity.

Other studies applied NIRS to assess coffee roasting degree and the changes in moisture, density and weight during roasting, but samples were scanned as ground material, thus losing the information related to possible within-batch variation (Alessandrini et al., 2008, Shan et al., 2015). Shan et al. (2015) demonstrated a promising prediction of roasting degree for a single Arabica coffee batch at increasing roasting time. The PLS regression had high R^2^ values, of 0.99 and 0.97 for the calibration and validation datasets, with root mean square error of cross-validation (RMSECV) of 0.81% (range: 2–18%). The spectral band at 1940 nm, mostly attributable to water, was described as the most important in the regression model. Alessandrini et al., 2008, Barbin et al., 2014 demonstrated prediction of roasting degree by PLS regression for a wider range of roasting conditions. The spectral bands at 1450 and 1943 nm had the most influence on the model, indicating the first overtone of O—H stretching and the combination band of O—H stretching and O—H deformation, respectively. NIRS was applied by Santos et al. (2016) to measure titratable acidity over roasting, reporting good prediction ability for bulk samples (R_p_^2^ = 0.89 and RMSEP = 0.16 mL NaOH 0.1 mol L^-1^ g^−1^ of coffee).

Many studies on coffee aroma have focused on ground material (Baqueta et al., 2019, Chang et al., 2021). Whilst it provides a good representative measurement of bulk properties, it does not provide information on heterogeneity. For example, Baqueta et al. (2019) used NIR spectroscopy on commercial ground coffee roasted at different roasting degrees, reporting promising results in terms of coffee cup quality.

Hyperspectral imaging (HSI) combines the non-destructive and fast nature of NIRS with imaging, providing spatially resolved spectra that enables sample heterogeneity to be studied. It has been applied to many other commodities (Caporaso et al., 2018a, Caporaso et al., 2018b, Caporaso et al., 2018) and high value foods (Ni, Liu, Liu, Sun, Pan, Fisk & Liu, 2020), but there have been relatively few studies for coffee beans. Fiore et al. (2008) used blends of Arabica and Robusta at four roasting degrees, and scanned the samples as ground coffee by HSI in the spectral region 400–1000 nm. Principal component analysis (PCA) showed good clustering between Arabica and Robusta green coffees, while medium and dark roasted samples could not be clearly differentiated. Linear Discriminant Analysis (LDA) was applied and 75–95% correct classification was achieved on ground roasted coffee to both differentiate the coffee species and the amount of Arabica in the blend. A study on the application of HSI for the assessment of whole roasted coffee beans to understand the consistency of roasted batches has been published by Nansen et al. (2016), using one batch of coffee only, roasted under different conditions and building a classification model to discriminate these roasting conditions (termed “roasting defects”), although no quantitative prediction of degree of roast was made. Chang et al. (2021) recently applied HSI to classify ground roasted coffee lots clustered into seven categories based on their flavour and reported an accuracy ranging from 70 to 77%.

HSI has been also applied to whole green coffee beans to study chemical composition (Caporaso et al., 2018a, Caporaso et al., 2018b, Nogales-Bueno et al., 2020) or to detect insect infestation (Chen, Chang, Ou, & Lien, 2020), however literature is lacking on its use to estimate aroma potential from roasted beans. Therefore, the aim of this paper was to determine, for the first time, the feasibility of HSI to predict coffee aroma by scanning roasted coffee beans on an individual coffee bean basis.

## 2 Materials and methods

### 2.1 Coffee samples and chemicals

Samples of commercial Arabica and Robusta green coffee were sourced from UK and European importers with the aim to obtain a wide geographical and botanical distribution of batches. A total of 25 coffee batches were used, and from each batch 10 beans were randomly selected. The batches were commercial samples from several growing locations, namely Brazil, Colombia, Costa Rica, Ethiopia, India, Mexico, Honduras, Kenya, Nicaragua, Uganda, Rwanda and Vietnam. The dataset included samples that had been treated using both the wet (∼60%) and dry (∼40% of the total batches) post-processing techniques, in order to include all possible variation expected on new real samples on the market. As soon as the samples were received, they were stored in a storage room at controlled temperature (∼10 °C) and humidity. These coffee beans represented the samples individually analysed in the current experiment. Reference chemical compounds for the volatile compounds analysed by GC–MS were obtained from Sigma-Aldrich (Steinheim, Germany), and Fluka (Buchs, Switzerland). The following compounds were used as standards for the GC–MS analysis: 2,3-pentanedione (97%), hexanal (98%), 1-methyl-1H-pyrrole (98%), 2-methyl-pyrazine (99%), 3-hydroxy-2-butanone, 2,5-dimethylpyrazine (98%), 2,6-dimethylpyrazine (98%), ethylpyrazine (98%), 2,3-dimethylpyrazine (99%), 2-ethyl-6-methylpyrazine (95%), 2-ethyl-5-methylpyrazine (98%), 2-ethyl-3-methylpyrazine (98%), acetic acid (99.5%), acetoxyacetone (98%), 2-ethyl-3,5-dimethylpyrazine (47.5%), 5-methylfurfural (99%), 2-furanmethanol (98%), 3-methyl-butanoic acid (98%), guaiacol (98%), 4-ethylguaiacol (98%) and 4-vinylguaiacol (98%).

### 2.2 Sample preparation and roasting

Samples were roasted using a Fracino Roastilino roaster (Fracino, Birmingham, UK). This is a fluid-bed type and was chosen as it gives uniform roasting conditions and was suitable for roasting of single beans. Preliminary tests were conducted to identify the best time–temperature profile to give a medium–high roasting level to all beans. This comprised running experiments in the time range 2–6 min and at 160–230 °C, always in isothermal conditions. Based on these trials, individual coffee beans were roasted using a standard time/temperature profile of 210 °C for 3 min. After roasting, beans were cooled, hyperspectral images were taken and the roasted coffee beans were then stored frozen at −20 °C until the point of grinding. The beans were removed from the freezer, ground using a manual grinder (Devo, Holland) and re-frozen for analysis in 1.5 mL Eppendorf tubes at −80 °C prior to SPME-GC–MS analysis. This second freezing step immediately after grinding was necessary to prevent the loss of compounds with high volatility before the GC–MS analysis.

### 2.3 Solid Phase microextraction – Gas Chromatography - Mass spectrometry of single coffee beans

Volatiles from individual beans were analysed by SPME-GC–MS. Coffee volatile compound sampling conditions followed the approach of Caporaso, Genovese, Canela, Civitella, and Sacchi (2014). The SPME fibre was a 1 cm 50/30 μm DVB/Carboxen/PDMS StableFlex (Supelco, Sigma Aldrich, UK) and was pre-equilibrated at 40 °C for 10 min. Fibre exposure was 20 min and injection time was 5 min. A trace 1300 series Gas-Chromatograph coupled with the Single-Quadrupole Mass Spectrometer (Thermo Fisher Scientific, Hemel Hemptead, UK) was used for analysis. The GC conditions were chosen based on Akiyama et al. (2007), slightly adapted to the type of column available. The column used was a 30 m length Zebron ZB-WAX capillary column (Phenomenex, Macclesfield, UK), with 0.25 mm internal diameter and 1.00 µm film thickness. The initial column temperature was 40 °C, which was held for 5 min, followed by a 3 °C min^−1^ temperature ramp to 180 °C, then a temperature ramp of 10 °C min^−1^ to 250 °C which was then maintained for 5 min. A constant carrier gas flow of 1.6 mL min^−1^ was applied. Mass spectra were acquired in the electron impact mode at 70 eV, using a *m*/*z* range of 50–650 with a 2 s scan time. Compound identification was conducted using reference compounds, where available, and by comparing the resulting mass spectra with a database (NIST). Identification was further confirmed by comparing linear retention indices (LRI) of volatiles under the experimental conditions reported above with literature data. The concentration of volatile compounds was expressed as the peak area as a proportion of the total GC–MS peak area. The quantification was carried out based on the internal standard used, while for those volatile compounds for which pure standards were available a proper quantification was carried out using calibration curves.

### 2.4 Gas Chromatography – Olfactometry of roasted coffee

For better identification of those volatile compounds that are more odour active, ground coffee (3 g) was placed into GC headspace vials (20 mL, 22.5 mm × 75.5 mm, Sigma-Aldrich, UK). 3-Heptanone was used as internal standard (15 μL, 0.01% 3-Heptanone (Sigma, Saint Louis, USA) in methanol (Laboratory reagent grade, Fisher Scientific, UK)) to calibrate for any instrument drift.

Aroma sampling conditions were conducted according to Liu et al. (2019). For GC-O nasal impact frequency (NIF) analysis, a splitter was fitted to the end of the ZB‐WAX column, and approximately half of the flow was diverted to an ‘odour sniffing port’ via a fused silica capillary passing within a heated transfer line, set at a temperature of 200 °C. Six panellists (four males and two females aged between 24 and 35 years) were used to conduct the GC-O NIF analysis. During each GC run, the panellist placed their nose close to the top of the sniffing port and recorded the time, duration and description of odours perceived (Pollien, Ott, Montigon, Baumgartner, Muñoz-Box, & Chaintreau, 1997). As the GC runs were 40 min long, two assessors were used to sniff each chromatogram, swapping over half‐way, to avoid fatigue.

### 2.5 Hyperspectral image acquisition, data treatment and statistical analysis

Roasted coffee beans were scanned on both sides using the HSI instrument described by Caporaso et al., 2018a, Caporaso et al., 2018b. Data for beans belonging to each batch were acquired in the same hypercube and treated as previously described by Caporaso et al. (2017).

The HSI system was supplied by Gilden Photonics Ltd. (Glasgow, U.K.), and the sensing technology was a SWIR spectral camera (Specim Ltd., Oulu, Finland) acquiring in the spectral range ∼ 900–2500 nm, with a spectral resolution of about 6 nm. The camera included a cooled 14 bit 320 × 256 pixel mercury‑cadmium-telluride (HgCdTe) detector and N25E spectrograph. The samples were placed on a black high density polyethylene tray whose movement was controlled by a stepper motor via the software at a speed of 10.9 mm s^−1^, and they were at a distance of 220 mm from the HSI camera. Illumination was provided by two 500 W halogen lamps. After each sample acquisition, a dark current image was acquired by closing the camera shutter and acquiring about 100 frames. At periodic intervals, a white reference was acquired by using a PTFE reference material with approximately 100% reflectance. SpectralCube 3.0041 software (Specim) was used for the acquisition, and statistical analysis of the single bean spectra was carried out using The Unscrambler (CAMO, Norway). PLS regression models were built for total content of volatile compounds and sensory scores; specifically, PLS2 was used, so that more response variables could be modelled, one for each volatile compound. A k-fold approach using 20 segments was used to select the samples to be used as the test dataset. To reduce the number of responses, volatile compounds were grouped according to either their chemical groups or their sensory descriptors from the literature, following our previous work aiming to understand the correlation of volatile compounds in roasted coffee, and thus identifying clusters to group them taking into account their chemical classes and sensory descriptors (Caporaso et al., (2018). Minitab 18 (Pennsylvania, USA) was also used to analyse the data of volatile compounds by PCA as a dimensionality reduction technique to understand possible sample grouping, and to perform correlation analysis using Pearson correlation, at a significance level of *p* < 0.05. Grouping according to the sensory descriptors would be similar to the creation of “aromatic series” or “odorant series” as recently reported for other food products (Genovese et al., 2019).

### 2.6 GC–MS analysis of batches of segregated coffee beans

As an additional test, a manual segregation trial was carried out to validate whether HSI predictions of coffee volatile aroma compounds or aroma profiles (predicted “nutty”, “roasted”, “sweet” and “spicy” scores) can be used to separate out individual beans into prototype production batches with distinctly different volatile aroma profiles. The HSI models developed to predict roasted coffee beans aroma were applied to one batch of Arabica and one batch of Robusta coffees as a further validation. Beans were roasted individually under the same conditions (3 min at 210 °C) of the calibration samples, and beans were scanned by HSI to then apply the calibration models. Beans were manually sorted into batches of beans with the highest 10% and lowest 10% of predicted values of the target attribute (volatile compound or analytically predicted sensory attribute). Success of segregation was evaluated by analysis of resultant batches by SPME-GC–MS as described previously.

## 3 Results and discussion

### 3.1 Reference analysis of volatile compounds

A total of 50 volatile compounds were identified by SPME-GC–MS in the roast and ground coffee samples (Table 1). Of the compounds identified, 26 were shown in this study to be key aroma compounds in coffee by GC-O and are annotated in the table, 11 had previously been shown in similar coffee systems to be odour active and the remaining 13 had been previously reported to be important in the volatile compound profile of coffee and were therefore also included. The main chemical classes of the compounds identified were ketones and pyrazines. In total there were 12 ketones, 12 pyrazines, 5 aldehydes, 4 phenols, 4 acids and a smaller number of other groups. The relative contribution of a volatile aroma compound to the overall flavour of coffee depends on its concentration, but also the individual detection threshold for the compound and its synergistic activity with other compounds (Caporaso et al., 2014, Genovese et al., 2014, Grosch, 1998, Grosch, 2001a, Semmelroch and Grosch, 1996), its volatility and ease of extraction from the matrix. Therefore, it is important to consider compounds from a broad range of coffee types when trying to understand the link between coffee chemistry and potential perceived flavour for a wide range of current and future applications.

**Table 1:** Performance of PLS2 regression model to predict individual volatile compounds in single coffee beans, by HSI scanning of roasted coffee beans.

Compound	Calibration		Cross-validation		RPD	Chemical class
	R^2^	RMSE	R^2^	RMSE		
acetaldehyde ^(2)^	0.220	0.160	0.120	0.170	1.37	Aldehyde
2-methylfuran ^(1A) (1R)^	0.313	0.092	0.249	0.097	1.13	Furan
3-methylbutanal ^(1A) (1R) (3)^	0.080	0.02	0.000	0.020	1.22	Aldehyde
2,3-butanedione ^(1A) (1R)^	0.285	0.065	0.188	0.070	1.16	Ketone
2,3-pentanedione ^(1A) (1R) (3)^	0.384	0.057	0.299	0.061	1.31	Ketone
hexanal ^(1A) (3)^	0.197	0.017	0.046	0.019	0.84	Aldehyde
1-methyl-1H-pyrrole ^(1A) (3)^	0.331	0.098	0.164	0.108	1.57	Heterocyclic N
pyridine ^(2)^	0.417	1.906	0.236	2.193	1.72	Heterocyclic N
pyrazine ^(1R) (2)^	0.608	0.416	0.479	0.482	1.27	Pyrazine
2-methyl-pyrazine ^(2) (3)^	0.689	1.841	0.552	2.222	1.48	Pyrazine
acetoin ^(1A)^	0.367	0.049	0.257	0.054	1.09	Ketone
acetol ^(4)^	0.427	0.346	0.319	0.379	1.27	Ketone
2,5-dimethylpyrazine ^(1A) (1R) (3)^	0.606	0.790	0.441	0.951	1.47	Pyrazine
2,6-dimethylpyrazine ^(1A) (1R) (3)^	0.646	0.843	0.512	0.992	1.43	Pyrazine
ethylpyrazine ^(1A) (1R)^	0.755	0.339	0.631	0.418	1.62	Pyrazine
2,3-dimethylpyrazine ^(3)^	0.378	0.235	0.229	0.263	1.08	Pyrazine
1-hydroxy-2-butanone ^(1A) (1R)^	0.484	0.028	0.378	0.031	1.34	Ketone
3-ethylpyridine ^(2)^	0.362	0.040	0.160	0.046	1.85	Heterocyclic N
2-ethyl-6-methylpyrazine ^(1A) (1R) (3)^	0.645	0.414	0.538	0.478	1.51	Pyrazine
2-ethyl-5-methylpyrazine ^(1A) (1R) (3)^	0.666	0.259	0.534	0.308	1.46	Pyrazine
2-ethyl-3-methylpyrazine ^(3)^	0.602	0.182	0.459	0.214	1.26	Pyrazine
2,3-diethylpyrazine ^(4)^	0.657	0.001	0.509	0.003	1.36	Pyrazine
3-ethyl-2,5-dimethylpyrazine ^(3)^	0.438	0.366	0.321	0.405	1.27	Pyrazine
acetic acid ^(1A)^	0.386	3.618	0.146	4.299	1.04	Acid
furfural ^(1A) (1R)^	0.642	1.928	0.520	2.242	1.59	Aldehyde
acetoxyacetone ^(4)^	0.630	0.081	0.542	0.091	1.53	Ketone
furfurylmethyl sulphide ^(4)^	0.390	0.036	0.320	0.038	1.39	Sulphide
2-ethyl-3,5-dimethylpyrazine ^(4)^	0.090	0.001	0.010	0.001	1.82	Pyrazine
furaneol ^(4)^	0.572	0.085	0.481	0.094	1.38	Ketone
2-acetylfuran ^(4)^	0.584	0.282	0.505	0.309	1.54	Furan
ethyl propanoate ^(3)^	0.651	0.074	0.580	0.082	1.54	Ester
2-furanmethanol acetate ^(1A) (1R) (3)^	0.565	0.262	0.518	0.276	1.74	Acetate
propanoic acid ^(2)^	0.576	0.241	0.470	0.270	1.34	Acid
5-methylfurfural ^(3)^	0.690	1.038	0.578	1.226	1.67	Aldehyde
2,3-butanediol ^(4)^	0.120	0.020	0.040	0.020	0.91	Alcohol
2-formyl-1-methylpyrrole ^(1R) (4)^	0.180	0.120	0.090	0.120	1.29	Pyrrole
γ-butyrolactone ^(4)^	0.200	0.080	0.100	0.090	1.13	Ketone
2-furanmethanol ^(1A)^	0.210	2.19	0.190	2.390	1.11	Alcohol
3-methyl-butanoic acid ^(1A)^	0.284	0.257	0.210	0.271	1.16	Acid
*N*-acetyl-4(H)-pyridine ^(1A) (1R)^	0.320	0.054	0.238	0.058	1.35	Heterocyclic N
3-hydroxy-4.5-dimethyl-2(5H)-furanone ^(4)^	0.598	0.053	0.529	0.057	1.43	Ketone
3-methoxy-5-methyl-2-cyclopenten-1-one ^(4)^	0.362	0.015	0.255	0.017	1.07	Ketone
3-methyl-2-butenoic acid ^(1A) (1R)^	0.443	0.002	0.333	0.002	1.26	Acid
3-methyl-1,2-cyclopentanedione ^(3)^	0.090	0.180	0.000	0.19	1.03	Ketone
guaiacol ^(1R) (3)^	0.559	0.161	0.486	0.175	1.87	Phenolic
2-(1H-pyrrol-2-yl)-ethanone ^(4)^	0.501	0.130	0.441	0.138	1.34	Ketone
2-formylpyrrole ^(4)^	0.446	0.213	0.352	0.231	1.30	Phenolic
phenol ^(1A) (1R)^	0.273	0.225	0.307	0.237	1.70	Heterocyclic N
4-ethylguaiacol ^(1A) (1R) (3)^	0.507	0.0005	0.434	0.0005	1.36	Phenolic
4-vinylguaiacol ^(1A) (1R) (3)^	0.483	0.118	0.423	0.126	1.25	Phenolic

The PLS2 regression model was built on the HSI spectra pre-treated using 2nd derivative treatment. The RMSE values are expressed as peak percentage (%) over the total peak area. The compounds are listed according to their GC elution order. Compounds identified with (1A) or (1R) are identified as key aroma compounds in coffee by GC-O as described in the methods section for Arabica or Robusta coffee respectively. Compounds numbered (2):Mahmud et al. (2020) and (3): Caporaso et al. (2018) are those considered as potent odorants in roasted coffee, based on literature data, and compounds numbered (4): Caporaso et al. (2018) have previously been identified in coffee and are important marker compounds.

Whilst many the compounds present are similar, it is known that there are differences between the relative abundance of volatile aroma compounds in Robusta and Arabica roasted coffee beans (Caporaso et al., (2018). Robusta is recognised to elicit more intense “roasted/smoky” and “sweet” sensory notes and in Arabica more “floral” notes are found. In our study, roasted Robusta coffee beans had a higher concentration of pyrazines, and a lower concentration of aldehydes, which would link to the expected sensory difference in the two coffee species.

### 3.2 HSI prediction of single volatile compounds from roasted coffee

Two approaches were tested to build prediction models for the concentration of volatile compounds in roasted coffee beans based on HSI scans of single roasted beans. Firstly, prediction models were built on all volatile compounds individually, while a second approach used a data reduction strategy to cluster compounds according to their chemical groups (aldehydes, ketones, pyrazines, acids, etc.), or according their odour impact, i.e. sensory descriptors (section 3.3).

Table 1 reports the results of the PLS regression models for individual volatile compounds in single roasted coffee beans by HSI, showing the coefficient of determination R^2^ and the root mean square error (RMSE) for the calibration and cross-validation datasets, as well as the ratio to performance deviation (RPD).

In general terms, compounds belonging to the pyrazine group had the best prediction performance compared to other chemical classes, with 2-methylpyrazine, ethylpyrazine, 2,6-dimethylpyrazine, 2-ethyl-6-dimethylpyrazine and 2-ethyl-5-dimethylpyrazine demonstrating R^2^ values in single roasted coffee beans of approximately 0.6–0.7 for the calibration dataset and above 0.5 for the cross-validation one. Similarly, furaneol, a ketone described in the literature with sweet and caramel odour notes, and furfural, organoleptically described as “sweet, woody, almond”, were successfully predicted with acceptable performance for screening purposes. Ethylpyrazine, ethylpropanoate, 5-methylfurfural, 2-methyl pyrazine, acetoxyacetone, 2-ethyl-5-methylpyrazine and 2-ethyl-6-methylpyrazine were the compounds showing the highest cross-validation R^2^ values.

The lowest RMSECV values were obtained for 2-ethyl-3,5-dimethylpyrazine, 4-ethylguaiacol, 2,3-diethylpyrazine and 3-methyl-2-butenoic acid. The prediction error by itself, however, is not a good indicator of which compound was predicted with the best performance, as the range of concentrations at which each compound was found should be also taken into account. For example, despite the low prediction error of hexanal, this compound was poorly predicted as it had an R^2^ value close to zero and RPD value of 0.84. The ratio of performance to deviation (RPD) value may therefore be a better indicator of the model performance, but it should still be considered alongside the calibration and prediction R^2^ values. The RPD is defined as the ratio of the model’s prediction error to the standard deviation of the reference target compound(s) and is often used as an indicator of how well a prediction model performs in relation to the observed variation of the analytes of interest. The individual models that had the higher RPD values were guaiacol, 3-ethylpyridine, 2-ethyl-3,5-dimethylpyrazine and 2-furanmethanol acetate. However, despite the high RPD values obtained for 3-ethylpyridine and 2-ethyl-3,5-dimethylpyrazine, these compounds were in fact poorly predicted (validation R^2^ of 0.16 and 0.01, respectively). The pyrazine compounds, with exceptions (2,3-dimethyl pyrazine and 2-ethyl-3,5-dimethylpyrazine), showed the best prediction performances. In addition, good prediction was obtained for guaiacol (RPD = 1.87), 2-furanmethanol acetate (RPD = 1.74), 5-methylfurfural (RPD = 1.67) and furfural (RPD = 1.59).

Additional Fig. 1 shows the plot of cross-validation error (RMSECV) in relation to the number of Latent Variables (LV), for all single volatile compounds. This graph is useful to display the expected prediction error and was used to select the optimal LV. The large difference in the prediction error among volatile compounds was attributed to the different concentrations at which they are found in coffee headspace, as some of them were present at 10–20% of total volatile GC area, while others were far below 1%. Some of the volatiles showing the best performances are shown as predicted versus reference plots in Additional Fig. 1. Generally, the concentration of all volatile compounds showed a good spread across the observed range, not displaying gaps and therefore the range of variation was fully covered.

It might seem that the number of optimal LVs used for some volatile compounds or some models were quite large. However, this number was optimised by cross-validation in order to avoid both under-fitting or over-fitting of the models. In the case we verified no significant improvement in terms of cross-validation error when increasing the number of LVs we chose the lowest possible one such that we optimised for model robustness rather than model’s performance and avoid overfitting.

The results obtained by HSI models for single volatile compounds showed that generally compounds with very similar chemical structure had similar performance, with exceptions, and similar strong absorbance bands as per their β-regression plots, despite differences in the concentration of the target volatile compounds.

### 3.3 Roasted coffee: Prediction of chemical groups and sensory impact

During roasting of complex food materials such as coffee, the kinetics and direction of roasting reactions are dependent on the presence and availability of flavour precursors. As each coffee bean differs, both in levels of these precursors, but also in available solvent (water) and microchemistry (pH), the resultant flavour compounds will also vary individually on a bean to bean basis. However, due to the complexity of the interacting reactions (Maillard chemistry, oxidation, pyrolysis, etc.) measuring and following each of these reactions simultaneously is almost impossible. Understanding how groups of compounds are formed, by each chemical class, will therefore offer insight into the overall rate of the different roasting reactions and grouping compounds by their predicted sensory attributes will offer insight into the resultant potential flavour differences (Caporaso et al., (2018). Clustering volatile compounds is also a strategy to reduce the number of responses in the statistical model thereby providing a more practical and usable model for commercial and research applications.

Individual volatile compounds were therefore grouped according to their chemical classes and new PLS regression models were built. This is presented in Table 2, which compares different spectral pre-processing techniques for HSI scans of single roasted coffee beans. The two spectral pre-treatments tested were standard normal variate (SNV) and 2nd derivative by the Savitzky–Golay algorithm. These spectral pre-treatments gave similar results, with the former resulting in slightly better prediction of phenols and heterocyclic nitrogen compounds. Although the prediction accuracy is lower than SNV in some cases, it uses fewer LVs, and thus might be preferred. Aldehydes and pyrazines were predicted with the highest accuracy among all chemical groups, showing R^2^ values of approximately 0.8 for calibration and 0.7 for cross-validation dataset.

**Table 2:** Results of PLS2 prediction of volatile compounds as chemical groups, using different spectral pre-treatment, on HSI spectra acquired on single roasted coffee beans.

Spectra pre-treatment	LV	Parameter	Calibration		Validation		RPD
			R^2^	RMSE	R^2^	RMSE	
Log(1/R)	17	Aldehydes	0.717	2.83	0.633	3.22	1.61
		Pyrazines	0.774	4.28	0.662	5.30	1.56
		Ketones	0.562	0.59	0.452	0.66	1.32
		Phenols	0.509	0.30	0.381	0.34	1.37
		Acids	0.375	3.83	0.208	4.36	1.10
		Heterocyclic N	0.385	2.58	0.221	2.93	1.26
		Aldehydes/Pyrazines	0.693	0.19	0.596	0.22	1.50
SNV	17	Aldehydes	0.793	2.33	0.701	2.77	1.87
		Pyrazines	0.807	3.61	0.726	4.39	1.88
		Ketones	0.600	0.55	0.497	0.60	1.45
		Phenols	0.544	0.26	0.439	0.29	1.61
		Acids	0.368	3.73	0.208	4.24	1.13
		Heterocyclic N	0.396	2.14	0.198	2.49	1.48
		Aldehydes/Pyrazines	0.750	0.17	0.666	0.19	1.74
2nd derivative	11	Aldehydes	0.776	2.42	0.676	2.85	1.82
		Pyrazines	0.808	3.65	0.699	4.65	1.78
		Ketones	0.556	0.57	0.433	0.64	1.37
		Phenols	0.589	0.23	0.542	0.25	1.87
		Acids	0.361	3.69	0.182	4.26	1.12
		Heterocyclic N	0.376	1.79	0.263	1.95	1.89
		Aldehydes/Pyrazines	0.769	0.16	0.669	0.19	1.74

LV = number of latent variables; R^2^ = coefficient of determination; RMSE = root mean square error; RPD = ratio to performance deviation; SNV = standard normal variate.

**Fig. 1:**
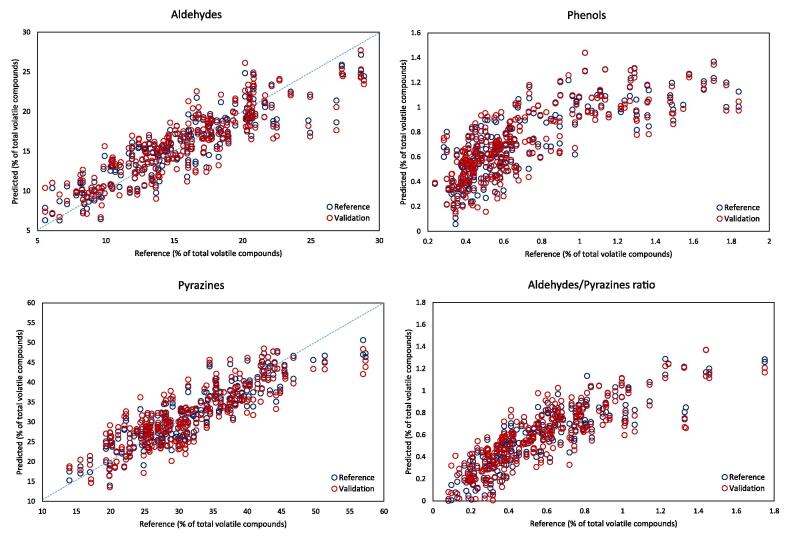
Predicted versus reference plots of some chemical groups predicted by HSI on single roasted coffee beans. Pre-treatment: 2nd derivative (LV = 11).

An additional aroma indicator is the ratio between aldehydes and pyrazines, as the former is linked to fruity and sweet notes, and the latter to roasted, nutty and smoky aroma. The ratio between aldehydes and pyrazines was also well predicted by HSI, with an RPD value of 1.7. The best RPD values for aldehydes and for pyrazines were 1.87 and 1.88, respectively, thus indicating sufficient accuracy for screening purposes. The RPD value for chemical groups was above 1.5 for pyrazines, ketones and the ratio aldehydes/pyrazines. Previous authors (Bellon-Maurel et al., 2010, Davey et al., 2009, Ncama et al., 2017) suggested that RPD values between 1.4 and 2 could be considered as fairly reliable, while for quantification, values above 2 are desired. The predicted versus reference plots for aldehydes, pyrazines, phenols and aldehydes/pyrazine ratio are shown in Fig. 1.

**Fig. 2:**
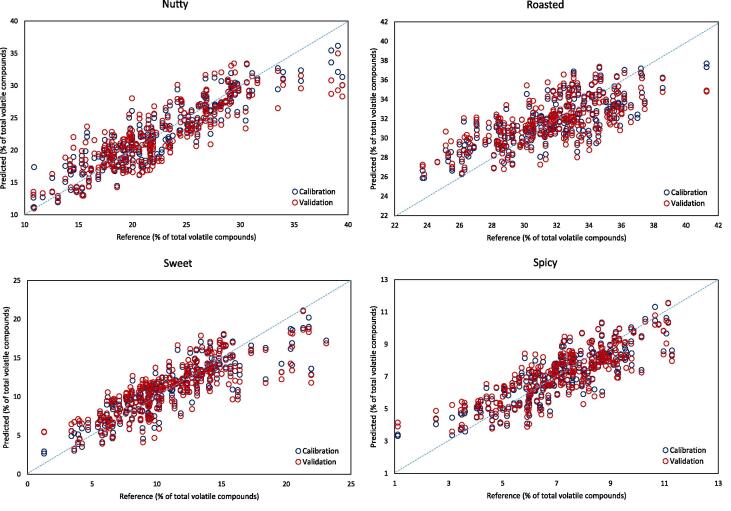
Predicted versus reference plots of some odour predictions by HSI on single roasted coffee beans. The spectral pre-treatment applied was 2nd derivative (LV = 11).

Table 3 reports the performance of the models built using the second approach for data reduction, grouping the volatile compounds into “sensory groups” according to the main odour descriptor for each compound. In this way, an indication of the possible sensory impact can be obtained. Spectral pre-treatments gave improvement for all the models tested, except “fruity” and “musty”, where little improvement was obtained. The descriptors predicted with the highest accuracy were those of “nutty”, “sweet” and “spicy”, which had calibration R^2^ values greater than 0.7, and 0.812 for nutty. The cross-validation R^2^ was 0.717 for nutty, and 0.63–0.64 for sweet and spicy. The poorest performance was for the fruity descriptor, for which the RPD value was below 1.0. Generally, lower performances were obtained for the prediction of odour impact in roasted coffee beans, compared to the use of chemical groups. The RPD values for sweet and spicy suggests the model has applicability for screening purposes (1.73 and 1.76, respectively). As observed in the case of chemical groups, the 2nd derivative pre-treatment gave a similar performance to SNV, while it made use of 11 LVs instead of 16. Although a large number of LVs were used, the models are not overfitted, as verified by checking the prediction error versus the number of LVs used. In addition, the model using the lowest number of LVs was preferentially used, in this case the one using the 2nd derivative treatment. SNV spectral pre-processing also led to slightly better calibration and validation errors compared to the 2nd derivative treatment. The predicted versus reference plots for the odour characteristics of nutty, roasted, sweet and spicy are shown in Fig. 2.

**Table 3:** Results of PLS2 prediction of volatile compounds as expected odour impact in terms of sensory odour descriptors from GC–MS analysis, using different spectral pre-treatment, by HSI acquired on single roasted coffee beans.

Spectra pre-treatment	LV	Parameter	Calibration		Validation		RPD
			R^2^	RMSE	R^2^	RMSE	
Log(1/R)	19	Fruity	0.410	0.426	0.212	0.496	0.90
		Nutty	0.725	2.949	0.573	3.69	1.47
		Roasted	0.549	2.168	0.368	2.583	1.23
		Sweet	0.721	2.20	0.589	2.692	1.50
		Sour	0.390	3.523	0.216	4.020	0.95
		Spicy	0.724	1.069	0.588	1.314	1.40
		Musty	0.647	2.698	0.513	3.192	0.68
		Positive/Negative	0.504	0.241	0.335	0.280	1.05
SNV	16	Fruity	0.380	0.35	0.206	0.39	1.15
		Nutty	0.799	2.432	0.707	2.998	1.81
		Roasted	0.604	2.000	0.448	2.245	1.42
		Sweet	0.757	1.983	0.648	2.334	1.73
		Sour	0.280	3.240	0.130	3.65	1.05
		Spicy	0.762	0.895	0.686	1.042	1.76
		Musty	0.466	1.590	0.329	1.783	1.22
		Positive/Negative	0.467	0.214	0.323	0.246	1.19
2nd derivative	11	Fruity	0.268	0.458	0.091	0.509	0.88
		Nutty	0.812	2.433	0.717	3.060	1.78
		Roasted	0.575	2.077	0.426	2.433	1.31
		Sweet	0.743	2.033	0.632	2.374	1.70
		Sour	0.335	3.250	0.130	3.768	1.02
		Spicy	0.729	0.984	0.637	1.155	1.59
		Musty	0.358	1.900	0.225	2.084	1.05
		Positive/Negative	0.490	0.220	0.319	0.259	1.14

LV = number of latent variables; R^2^ = coefficient of determination; RMSE = root mean square error; RPD = ratio to performance deviation; SNV = standard normal variate. The reference values for these aroma descriptors were derived from the GC–MS data, by grouping individual volatile compounds according to their odour descriptors (Caporaso et al., 2018a). The value “Positive/negative” indicates the fraction of volatile compounds reported with positive descriptors over those reported in the literature with negative descriptors.

### 3.4 SPME GC–MS validation of coffee bean segregation

**Fig. 3:**
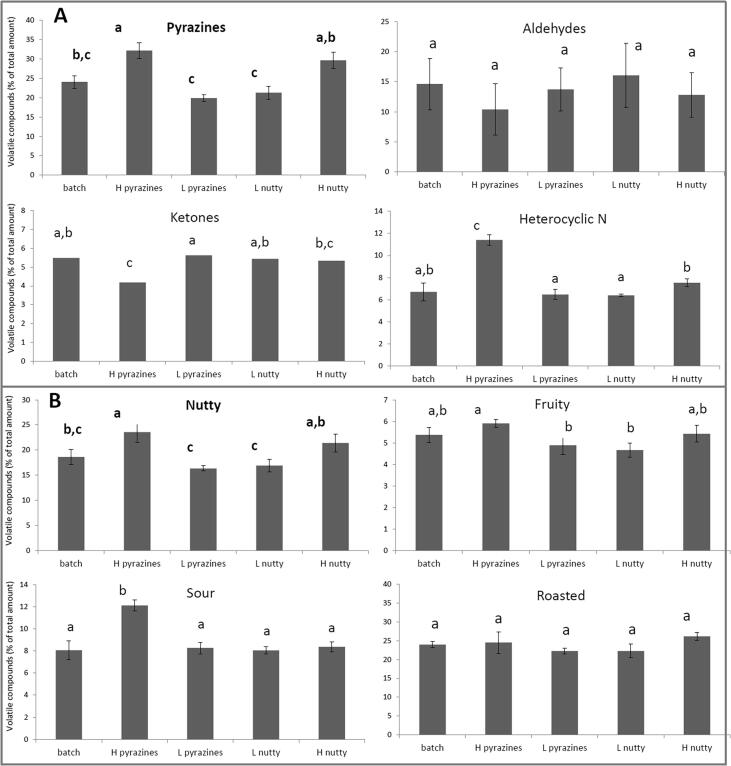
Impact of coffee bean segregation trials after sorting beans for the top 10% (H) or lowest 10% (L) concentration of A) predicted pyrazines or B) analytically predicted “nutty”, indicated in bold, on the relative abundance of 4 groups of volatile compounds (pyrazines, aldehydes, ketones and heterocyclic nitrogen) and analytical predicted “nutty”, “fruity”, “sour” and “roasted”. Primary segregation targets are highlighted in bold. Different letters indicate a statistically significant difference among the samples by ANOVA, *p* < 0.05, Tukey's HSD test.

As an example of the capability of the HSI approach, a single batch of Arabica coffee beans was roasted and HSI models applied for A) pyrazines and B) analytically predicted “nutty”. Beans were then manually segregated according to the prediction (high and low) and the resultant sorted batches analysed by GC–MS and directly compared to the residual material (Fig. 3).

Sorting for predicted pyrazine content (High Pyrazine, HP) resulted in a batch of roasted coffee beans with an increased concentration of pyrazines compared to the original “batch” material, this difference was statistically significant (p < 0.05) (Fig. 3A). Total pyrazines increased from 24.63 % ± 1.46 % to 32.72 % ± 2.57 % of total volatiles measured (Fig. 3A). Sorting for pyrazines led to a reduction in ketones and an increase in heterocyclic nitrogen-based compounds (p < 0.05). There was also an increase in analytically predicted “nutty” and analytically predicted “sour” (p < 0.05) (Fig. 3B).

Sorting for analytically predicted “nutty” resulted in two batches of roasted coffee beans which, whilst not statistically different to the reference material, were significantly different from each other, having higher analytically predicted “nutty” and lower analytically predicted “nutty” (Fig. 3B). The segregated batches also contained significantly different concentrations of pyrazines and heterocyclic nitrogen (p < 0.05) (Fig. 3A).

### 3.5 Discussion

The results shown in this paper demonstrate the successful prediction of volatile compounds from single coffee beans by HSI and demonstrate its application for screening purposes. Differences in the prediction ability were obtained even for compounds with very similar chemical structures, and the relative concentration was not a critical factor in terms of prediction ability. This is explained not only by the chemical structure of the molecules, but it is more likely linked to the biochemical origin of these volatile compounds. Generally, compounds belonging to the pyrazine class showed the strongest prediction. Pyrazines are heterocyclic aromatic compounds that are described as nutty, roasted, peanut, coffee-like and earthy, and many pyrazines are described as potent odorants in roasted coffee (Czerny and Grosch, 2000, Grosch, 2001b, Scheidig et al., 2007). The similarity of some models predicting specific volatile compounds could be attributed to the common origin of these compounds, which might explain the better performance of models obtained by grouping compounds belonging to the same chemical class.

The regression vectors, i.e. the coefficients of the PLS regression models, for aldehydes and pyrazines shared the most important absorption peaks and were negatively correlated. Some of the most intense absorption wavelengths for several models were attributed to the 1st overtone of O—H and N—H, which was previously reported to be important wavelengths for the prediction of “bitterness” in coffee assessed by sensory analysis (Ribeiro, Ferreira, & Salva, 2011). Absorption wavelengths previously associated with the overall “flavour” were those around 1975 and ∼ 1435 nm, while those at 2276 and 2088 nm were important for acidity and bitterness, respectively (Ribeiro et al., 2011). Spectral bands at ∼ 2050 nm likely show the absorbance of the 1st overtone of C=O and O—H combination; those around ∼ 2300 nm have been described as due to N—H and O—H combination or C—H combination. 1680 nm is likely attributed to the 1st overtone of C—H, while the region 1950–1980 nm is related to the 2nd overtone of C=O stretching. The absorption around 2000–2100 nm is due to both 1st overtone of C=O and O—H combination bands, while the region 1400–1450 nm shows the 1st overtone of O—H and N—H.

The understanding of coffee quality using vibrational spectroscopy methods is challenging due to the low concentrations of compounds that exert a sensory impact, in terms of both aroma and taste. Some taste-active compounds are found at relatively high concentrations, examples of which would include acids and caffeine in roast and ground coffee (Farah, 2012), and it therefore might be possible to detect them directly by NIR. In contrast, the concentration of volatile compounds is relatively low and therefore unlikely to be directly predicted. For these reasons, other authors have previously attempted to build NIR models to predict some sensory characteristics of the coffee beverage instead of quantifying specific compounds. Ribeiro, Ferreira, & Salva (2011) applied cup testing to bulk ground and roasted coffee and built NIR models (1100–2500 nm) to predict sensory parameters such as acidity, bitterness, flavour, cleanliness, body and “overall quality” while Esteban-Díez et al. (2004) reported PLSR models for the sensory attributes of body, acidity, bitterness and appearance. Both of these studies webre carried out on batches of roast and ground coffee (i.e. not single coffee bean), which has limitations that were overcome in our research as we have demonstrated that volatile compounds that represent coffee aroma can be predicted for individual beans with sufficient accuracy to allow screening.

Ultimately, the application of this insight could enable better standardisation of roast coffee quality, removal of defect beans and even selection of the most distinctive batches of coffee beans for special target markets.

Previous research has shown the possibility of predicting coffee aroma using NIR-based techniques, whilst more limited research has been done using HSI. Previous authors have applied NIR spectroscopy to predict coffee cup quality (Tolessa, Ramaker, De Baets & Boeckx, 2016), or the roasting degree (Alessandrini et al., 2008) as well as the ratio between Arabica and Robusta (Bertone, Venturello, Giraudo, Pellegrino & Geobaldo, 2016), or the sensory properties of the brewed coffee (Esteban-Díez et al., 2004). A recent book chapter Baqueta, Caporaso, Coqueiro & Vanderrama, (2020) describes the potential of NIR spectroscopy for coffee quality evaluation and describes some successful research carried out for coffee cup classification. Regarding coffee aroma analysed by sensory panels showed promising results by using PLS on NIR data obtained on ground coffee lots even using miniaturised NIR instruments (Baqueta et al., 2019), however applications at a single coffee bean level or using HSI to predict for coffee aroma are lacking.

A recent review summarises the current research related to predicting food minor compounds using these techniques, including volatile compounds (Tahir et al., 2019). The authors reported some applications of conventional NIR spectroscopy to predict volatile compounds in olive oil, wine and lavender oil, as well as one publication on cheese, while only three research papers attempted to predict minor constituents in food products, i.e. anthocyanin in wine, total phenolic content in grapes, and total phenolic content in cocoa beans, while no research has been conducted to investigate volatile compounds. This confirms the novelty of the research herein presented.

By comparing the outcome of our research with previous publications based on NIR spectroscopy but on other food products, we can state that HSI has a similar performance as it leads to similar values of regression coefficients and prediction error values. However, a direct comparison cannot be made due to the obvious influence of the other compounds in those food matrices. Generally, however, whilst this approach seems promising, it is likely due to the detection of biochemical precursors or intermediates found at a higher concentrations, or to some secondary correlation among compounds rather than an direct quantification of specific volatile compounds.

Future research on the topic could investigate the following: i) Selection of the most influential spectral bands in order to build multi-spectral imaging models and to verify whether and to what extend the prediction ability changes; ii) Application of these models to new coffee batches, including those from new geographical origins or different coffee species to understand whether the findings can be generalised or whether it is more appropriate to use species-specific calibrations; iii) investigation of the general ability of HSI or other vibrational spectroscopy technologies to differentiate and quantify volatile compounds; iv) investigation of the potential difference that different brewing techniques can bring to the brewed coffee (Caporaso et al., 2014) and build models the brewed coffees.

## 4 Conclusions

We have presented effective hyperspectral imaging prediction models for roast coffee aroma on a single roasted bean basis. To the best of the authors’ knowledge, this is the first study to achieve this. Predictions have been presented for single volatile compounds, compounds grouped by chemical class and compounds grouped by their analytically predicted odour properties. The predictive ability of the model was validated by segregating beans into batches (top 10% and bottom 10%) based on HSI predictions of groups of volatile compounds (pyrazines) and analytical predicted sensory traits (nutty). The resultant batches of beans were shown by SPME-GC–MS to have significantly different profiles of volatile flavour compounds and analytically predicted odour properties. This work demonstrates that the inherent variations in chemical profiles of individual coffee beans can be exploited, and by using HSI as a non-destructive evaluation tool, individual beans can be segregated, thereby producing batches of coffee with unique and distinct blends of volatile flavour compounds. This is of relevance to the coffee industry as it will provide new tools for quality evaluation, opportunities to understand and minimise heterogeneity during production and roasting processes and ultimately provide the tools to define and achieve new flavour profiles of coffee.

## CRediT authorship contribution statement

**Nicola Caporaso:** Conceptualization, Methodology, Investigation, Formal analysis, Writing – original draft. **Martin B. Whitworth:** Conceptualization, Supervision, Methodology, Software, Writing - review & editing. **Ian D. Fisk:** Conceptualization, Funding acquisition, Supervision, Writing - review & editing.

## Declaration of Competing Interest

The authors declare that they have no known competing financial interests or personal relationships that could have appeared to influence the work reported in this paper.
