# Supplementary material for: Prediction of coffee aroma from single roasted coffee beans by hyperspectral imaging
Source: Food Chem. 2022 Mar 1;371:131159. doi: 10.1016/j.foodchem.2021.131159 (PMC8617352; doi:10.1016/j.foodchem.2021.131159)
Supplement: Supplementary data 1 [file mmc1.docx]

Additional Figure 1. Prediction error (RMSECV) plot depending on the number of Latent Variables (LV) in PLS2 models to estimate individual volatile compounds in single roasted coffee beans using HSI (roasted beans were scanned). The optimal LV identified for the general model was LV=11.

Additional Figure 2. Predicted versus reference concentration of single volatile compounds (odour impact compounds) in single roasted coffee beans by using HSI, and PLS2 as the chemometric method. Compounds are expressed as % of each compound compared to the total GC-MS peak areas (n~300).
